# A Comprehensive Toxicological Safety Assessment of Superoxide Dismutase as a Novel Food Ingredient

**DOI:** 10.1155/jt/5535992

**Published:** 2026-10-29

**Authors:** Disi Chen, Xianghong Ke, Xiaoya Du, Qiwen Huang, Hongxin Peng, Qing Yu, Jie Tian, Ying Xia, Jingjing Qu, Yingjing Zhang, Bolin Fan, Wei Zhou

**Affiliations:** ^1^ College of Animal Science & Technology College of Veterinary Medicine Huazhong Agricultural University Wuhan 430070 China hzau.edu.cn; ^2^ Hubei Provincial Center for Disease Control and Prevention NHC Specialty Laboratory of Food Safety Risk Assessment and Standard Development Wuhan 430079 China cdc.gov; ^3^ China National Research Center for Food Safety Inspection and Testing (China National Research Center for Food Safety HACCP Application) Beijing 100045 China

**Keywords:** genotoxicity, oral toxicity, safety evaluation, superoxide dismutase, teratogenicity

## Abstract

This study systematically evaluated the toxicological safety of superoxide dismutase (SOD) as a novel food ingredient. Acute oral toxicity testing revealed no toxicity or mortality in mice of both sexes following a single gavage dose of 10 g/kg BW, classifying SOD as a practically nontoxic substance. In the 90‐day subchronic toxicity study, Wistar rats orally administered doses of 8.33, 25, or 75 mg/kg BW/day exhibited the following: Female rats in the high‐dose group showed significantly increased body weight during Weeks 11–13, transient fluctuations in food efficiency occurred in the low‐dose group, and high‐dose males displayed reduced neutrophil percentage (NEUT%) at termination, whereas all observed alterations in hematological, clinical chemistry, and urinalysis parameters lacked dose dependency and were largely disappeared during the recovery period. Histopathological examination revealed no substance‐specific lesions. Teratogenicity testing demonstrated a significant increase in fetal resorption rates in the 8.33 and 25 mg/kg BW groups (*p* < 0.01), whereas this effect showed no dose–response association and no structural malformations were observed. Three genotoxicity assays (mammalian erythrocyte micronucleus, chromosome aberration in vitro, and Ames tests) yielded negative results. Collectively, these findings demonstrate a favorable safety profile for SOD at the tested doses, with no evidence of genotoxicity either in vivo or in vitro.

## 1. Introduction

Superoxide dismutase (SOD) is a crucial antioxidant enzyme ubiquitously present in living organisms. It catalyzes the dismutation of the superoxide radical (O) into hydrogen peroxide (H_2_O_2_) and oxygen (O_2_) [1], playing an indispensable role in maintaining intracellular reactive oxygen species (ROS) homeostasis, protecting biomacromolecules (e.g., DNA, proteins, and lipids) from oxidative damage, regulating cellular signaling, and mitigating cellular aging processes [2, 3]. Leveraging its potent enzymatic antioxidant properties, SOD exhibits broad therapeutic potential in medicine, including as a potential therapeutic or adjunct agent for inflammatory diseases [4], radiation injury [5], organ transplantation protection [6, 7], neuroprotection [8], and antiaging interventions [9, 10]. In agriculture, SOD functions as a feed additive, significantly enhancing the growth performance and disease resistance of animals [11, 12]. Within the cosmetics industry, SOD is commonly incorporated as an antioxidant enzyme and antiaging ingredient, and its safety has been validated at concentrations up to 4% in both leave‐on and rinse‐off products [13]. In the food industry, SOD application primarily focuses on its potential as a natural antioxidant enzyme to delay oxidative deterioration, preserve food color and flavor, and extend shelf‐life [14–16]. Furthermore, SOD holds significant promise as a dietary supplement in beverages, health products, and functional foods for disease prevention and health promotion [17, 18].

It should be noted, however, that natural SODs generally exhibit limited stability during food processing and digestion, which has historically constrained their industrial applications [19]. Nevertheless, the Cu/Zn‐SOD evaluated in this study was specifically engineered through site‐directed mutagenesis to enhance its stability. Previous studies have demonstrated that modified SODs can maintain stability across a broad pH range (2.0–10.0) and at temperatures up to 80°C, supporting their potential as food ingredients [20]. The present study focuses on the toxicological safety assessment of this engineered SOD to provide a scientific foundation for its potential food applications.

Although Carillon et al. conducted a short‐term toxicological evaluation of SODB [21], the comprehensive safety assessment of SOD as a novel food remains incomplete, particularly regarding its toxicity under conditions of high‐dose, long‐term exposure. Both the European Union Novel Food Regulation [22] and China’s Administrative Measures for Safety Review of New Food Materials [23] mandate thorough toxicological evaluation for ingredients lacking a history of traditional consumption. This evaluation encompasses endpoints such as acute oral toxicity, skin sensitization, and genotoxicity. This study is based on the OECD Guidelines for the Testing of Chemicals [24] and the National Food Safety Standard (China) [25] to evaluate the safety of SOD as a novel food ingredient through acute oral toxicity, 90‐day subchronic toxicity, teratogenicity, and genotoxicity testing. The aim is to provide a scientific foundation for its compliant application in Chinese and global markets.

## 2. Materials and Methods

### 2.1. Test Materials

The SOD used in this study specifically referred to recombinant modified human Cu/Zn‐SOD (rmh Cu/Zn‐SOD) was supplied by Hubei Protein Biotech Co., Ltd. (Jingzhou, China). It has been reported in the literature that the chloroplast Cu/Zn‐SOD of *Withania somnifera* (GenBank: ADM86714) exhibits excellent stability and is particularly capable of withstanding high temperatures, acidic environments, and the action of trypsin [26]. We found that the number of variable residues, namely, lysine (K) and arginine (R), in its amino acid composition was much lower than that of human Cu/Zn‐SOD. In view of this, we developed a technical solution for a recombinant modified human Cu/Zn‐SOD (rmh Cu/Zn‐SOD) amino acid sequence based on the amino acid sequence of human Cu/Zn‐SOD (GenBank: AY450286), referring to the amino acid sequence of *Withania somnifera*’s chloroplast Cu/Zn‐SOD and those of other biological species. The resulting SOD product has significantly improved water solubility and enzyme activity, and has stronger tolerance to acidic environments and trypsin (Table 1) [27]. The preparation procedure involved prokaryotic expression of the gene in *Escherichia coli* BL21, followed by high‐pressure homogenization, centrifugation, hydrophobic interaction chromatography, buffer exchange via hollow fiber diafiltration, and lyophilization to obtain the final lyophilized powder, and the specific steps for protein purification can be referred to in previous work [28]. The final product demonstrated high purity (> 90%) and a specific activity > 8000 U/mg. This manufacturing process was successfully scaled for industrial production.

**TABLE 1:** Mutation method of rmh Cu/Zn‐SOD amino acid sequence.

Number	Site^∗^	*Withania somnifera* SOD	Human SOD	rmh Cu/Zn‐SOD	Nucleotide coding
1	24	—	K	Q^∗∗^	CAG
2	31	T	K	T	ACC
3	37	T	K	T	ACT
4	70	L	R	L	CTG
5	71	T	K	T	ACC
6	76	G	K	G	GGC
7	92	A	K	A	GCC
8	123	L	K	L	CTG
9	137	T	K	T	ACC

^∗^Numbering and locating according to the human Cu/Zn‐SOD amino acid sequence.

^∗∗^Determined by referencing the corresponding amino acid residues in organisms such as chickens and fruit flies.

### 2.2. Chemicals

All chemical reagents, solvents, and other chemicals used in the experiments were of analytical or chemical grade and were obtained from commercial sources.

### 2.3. Animals, Cells, and *Salmonella typhimurium* Strains

SPF‐grade KM mice and Wistar rats were provided by Hubei Provincial Experiment Animal Research Center and fed in the SPF animal house of Hubei Provincial Center for Disease Control and Prevention. The animal room temperature was consistently maintained at 20°C–26°C, and the relative humidity range was 30%–70% with 12‐h light/dark cycle. After all animals were quarantined, they were acclimatized to the laboratory environment for at least 3 d. During the experiment period, all animals were freely provided with distilled water and solid feed (Wuhan Wanqian Jiaxing Biotechnology Co., Ltd.). All animal experiments and procedures were approved by the Animal Management and Use Committee of the Hubei Food and Drug Safety Evaluation Center (Approval No. 202422139). Chinese hamster ovary cell strain (CHO‐K1) and *S. typhimurium* strains were obtained from the Chinese Center for Type Culture Collection and USA Moltox Company, respectively. This study adopted the method of Tian et al., and the methods’ description partly referenced their wording [29].

### 2.4. Acute Oral Toxicity

An acute oral toxicity test was performed according to the “National Food Safety Standard Acute Oral Toxicity Test: GB15193.3‐2014 (China)” [30]. Due to the lack of relevant literature reporting the toxicity of the test substance, this study referred to the limit test method outlined in the same standard. Based on a preliminary test, the maximum gavage concentration of the test substance was determined to be 0.5 g/mL, with a single gavage volume of 20 mL/kg BW. Therefore, the test substance was administered at a dose of 10.0 g/kg BW once daily for 14 consecutive days. A total of 20 KM mice (18–22 g, 10 females and 10 males) were included in the study. Mice were fasted for 4 h prior to oral gavage, and feed was supplied ad libitum after oral gavage. The animals were monitored individually for changes in general behavior, signs of toxic symptoms, and mortality at 30 min, 1, 2, 4, 6, and 10 h after initial administration and at least once daily for the next 14 days. The body weight of each animal was recorded before dosing, on Days 7 and 14, respectively. At the end of the observation period, all animals were sacrificed and prepared for gross pathological examinations.

### 2.5. Subchronic Toxicity

A total of 80 Wistar rats were randomly divided into 4 main groups, with 10 females and 10 males in each group, including a negative control group and three experimental groups, i.e., the low‐dose (8.33 mg/kg BW), mid‐dose (25 mg/kg BW), and high‐dose (75 mg/kg BW) groups, with a single gavage volume of 20 mL/kg BW (dissolved in distilled water). Simultaneously, 20 Wistar rats were set up to form two satellite control groups: a negative control satellite group and a high‐dose satellite group, with 5 females and 5 males in each group.

The rats in both main and satellite groups were administered SOD or distilled water by oral gavage for 90 d (6 d a week), and satellite groups continued to be observed for an additional 28 d to evaluate the reversibility or persistence of any treatment‐related effects. Eye examinations were performed at the beginning and end of the treatment period. During the treatment period, clinical observation was performed once a day, body weights and food intake were recorded every week, and average weekly food consumption was calculated for each animal. Urine and blood samples of individual rats were collected in 13th week in the main groups, on Day 45 (mid‐term) and Day 120 (recovery phase) in the satellite groups. Urine parameters were measured by an automatic urine analyzer (DIRUI H‐800, China), including the determination of the levels of glucose (GLU), protein (PRO), occult blood (BLD), pH, specific gravity (SG), bilirubin (BIL), urobilinogen (UBG), and ketone (KET). Hematology parameters of blood were measured by an automatic blood analyzer (Sysmex XT‐2000i, China), including red blood cell (RBC), white blood cell (WBC), lymphocyte (LY), monocytes (MO), neutrophil (NEUT), eosinophil (EO), basophil (BASO), platelet count (PLT), hematocrit (HCT), and hemoglobin (HGB). Clinical biochemistry parameters were analyzed using an automatic clinical chemistry analyzer (Beckman AU 680, USA), including the levels of alanine aminotransferase (ALT), aspartate aminotransferase (AST), total protein (TP), albumin (ALB), alkaline phosphatase (ALP), gamma‐glutamyl transferase (GGT), GLU, urea nitrogen (BUN), creatinine (Crea), total cholesterol (CHO), triglyceride (TG), potassium ion (K^+^), sodium ion (Na^+^), and chloride ion (Cl^−^). Activated partial thromboplastin time (APTT) and prothrombin time (PT) were assayed by automated coagulation analyzer (Mindray C3510, China). At the end of the experiment, a complete gross necropsy was performed for all groups of animals. During necropsy, the selected organs and glands, including brain, heart, liver, kidney, spleen, uterus/epididymis, ovaries/testis, thymus, and adrenal glands, were isolated and weighed separately. The following tissues and organs were fixed with 10% formalin, sliced, visualized with H&E staining, and underwent pathological examination: adrenal glands (bilateral), lungs, spleen, brain, lymph node, stomach, epididymis (bilateral), ovaries (bilateral), testis, heart, pancreas, thymus, kidneys (bilateral), hypophysis, thyroid and parathyroid glands, large intestine (cecum, colon, and rectum), prostate, bladder, liver, small intestine (duodenum, jejunum, and ileum), and uterus. The test was conducted in accordance with the “National Food Safety Standard 90 Days Oral Toxicity Test: GB 15193.13‐2015 (China),” and histopathological examinations were performed simultaneously [31].

### 2.6. Teratogenicity Test

A total of 104 females (210–340 g) and 52 males (360–500 g) Wistar rats were used for this study. Female and male rats were mated at a ratio of 2:1, and the successfully mated females were randomly assigned to 4 groups, including the negative control group and three dose groups: low (8.33 mg/kg BW), mid (25 mg/kg BW), and high (75 mg/kg BW). The positive control was referred to a previous teratogenic test in our laboratory within one year. Rats in each dose group were administered daily for 10 consecutive days from the 6th to 15th day of pregnancy by oral gavage with a volume of 10 mL/kg BW. Rats in the negative control group were gavaged with the same volume of distilled water. Rats were observed daily for general clinical condition, behavior, and mortality. Body weights of the pregnant female rats were measured on Days 0, 6, 9, 12, 15, and 20, with Day 0 (d0) defined as the day when sperm was first detected in the female vagina. On Day 20, the uterus was collected and weighed, and the number of corpora lutea, percent of implantation, live and dead fetuses, stillbirths, and fetal absorptions, fetal body length, weight, gender, and general appearance of teratogenicity were recorded. Live fetuses were randomly divided into two groups for skeletal and visceral examinations, respectively. This test was conducted in accordance with the “National Food Safety Standard Teratogenicity Test: GB 15193.14‐2015 (China)” [32].

### 2.7. Genetic Toxicity

#### 2.7.1. Mammalian Erythrocyte Micronucleus Test

Based on the results of the acute oral toxicity test, the LD50 of the test substance is greater than 10.0 g/kg BW. Its maximum gavage concentration is 0.5 g/mL; therefore, the high dose for the micronucleus test was set at 10.0 g/kg BW, with medium and low doses at 5.0 and 2.5 g/kg BW, respectively. Additionally, a negative control group (distilled water) and a positive control group (cyclophosphamide, 40 mg/kg BW) were included. The gavage volume was 20 mL/kg BW. A total of 50 KM mice (25–30 g) participated in the study, with 5 males and 5 females per group.

All groups were administered twice by oral gavage within 24 h. Mice were sacrificed 6 h after the second administration, and the femur bone marrow was collected for smears, dried naturally, fixed with methanol, stained with Giemsa, and observed under a microscope. A total of 2000 polychromatic erythrocytes (PCEs) for each mouse were observed, and the number of PCEs with micronuclei was recorded to determine the incidence of micronuclei. Simultaneously, a total of 200 PCEs and normochromatic erythrocytes (NCEs) were counted to calculate the percentage of PCEs in total erythrocytes (PCE + NCE). This test was conducted in accordance with the “National Food Safety Standard Micronucleus Test of Erythrocytes in Mammals: GB 15193.5‐2014 (China)” [33].

#### 2.7.2. Chromosome Aberration Test of Mammalian Cells In Vitro

Test substance dose groups were set at 5000 (+S9/−S9), 2000 (+S9/−S9), and 800 μg/mL (+S9/–S9), respectively. The negative control group received distilled water (+S9/−S9). The positive controls were provided with 15.0 μg/mL cyclophosphamide monohydrate (+S9) or 0.8 μg/mL mitomycin C (−S9). For +S9 groups, the S9 mix was added to a final concentration of 0.1 mL/mL, while no S9 mix was added to −S9 groups. Each culture flask received 5.0 mL of serum‐free DME/F‐12 medium containing either the negative control, positive control, or the respective concentration of test substance, according to the assigned groups. After gentle mixing, culture flasks were incubated in a CO_2_ incubator for 4 h (+S9/−S9) and 24 h (−S9), respectively. Following treatment, cells were rinsed appropriately and then replenished with complete medium containing 10% FBS, followed by culture at 37.0°C under 5% CO_2_ for 24 h until cell harvest. Approximately 4 h prior to harvest, colchicine was added to a final concentration of 1 μg/mL. Cells were then harvested using standard procedures involving trypsinization, hypotonic treatment, fixation, slide preparation, and staining. For each dose group, 200 well‐spread metaphase cells were analyzed. Chromosome aberration types and numbers were recorded, and the cell aberration rate was calculated (with chromatid gaps and polyploidy recorded separately and excluded from aberration rate statistics). This test was conducted in accordance with the “National Food Safety Standard Chromosome Aberration Test of Mammalian Cells In Vitro: GB 15193.23‐2014 (China)” [34].

#### 2.7.3. Bacterial Reverse Mutation Test

An Ames test was conducted using five strains of *Salmonella typhimurium* (TA97a, TA98, TA100, TA102, and TA1535). The test substance was evaluated at five dose levels per experiment (128, 320, 800, 2000, and 5000 μg/plate), alongside a spontaneous revertant control, a negative solvent control, and positive controls. Fenaminosulf (50 μg/plate) served as the −S9 positive control for strains TA97a, TA98, and TA102; sodium azide (2.0 μg/plate) was used as the −S9 positive control for strains TA100 and TA1535; 2‐aminofluorene (10 μg/plate) was the +S9 positive control for strains TA97a, TA98, and TA100; 1,8‐dihydroxyanthraquinone (50 μg/plate) was the +S9 positive control for strain TA102; and cyclophosphamide monohydrate (100.0 μg/plate) was the +S9 positive control for strain TA1535. For each strain and control group, three replicate plates were prepared per concentration. For each plate, 0.1 mL of the test strain suspension, 0.1 mL of the test substance solution (or control solution), and 0.5 mL of S9 mix (when metabolic activation was required) were added to the top agar and poured onto the bottom culture medium of the plates. The plates were incubated at 37°C for 48 h, and then, the number of revertant colonies per plate was counted. If the number of revertant colonies of the test substance is more than twice that of the untreated control group (spontaneous revertant group) and exhibits a dose–response relationship, it is considered positive. The entire experiment was repeated once, with only the dose settings changed (8, 40, 200, 1000, and 5000 μg/plate), while other conditions remained unchanged (Supporting Table S1). This test was conducted in accordance with the “National Food Safety Standard Bacterial Reversion Mutation Test: GB 15193.4‐2014 (China)” [35].

### 2.8. Data Analysis

Experimental data were presented as mean ± standard deviation. Statistical analysis was performed using SPSS software. Measurement data were analyzed using ANOVA for comparisons of means between multiple experimental groups and the control group. Categorical data were analyzed using nonparametric tests (chi‐square). All statistical analyses were stratified by gender of the animals.

## 3. Results

### 3.1. Acute Oral Toxicity

After administering the test substance by gavage, the mice showed no evident signs or symptoms of poisoning. During the 2‐week observation period, no animals died, and at the end of the experiment, no abnormalities were found during the gross anatomical examination of the mice.

According to the GB 15193.3‐2014 acute toxicity grading evaluation standard [30], the test substance was classified as practically nontoxic.

### 3.2. 90‐Day Oral Toxicity

#### 3.2.1. Daily Observation and Ophthalmic Examination

Throughout the observation period, the general condition of the animals in each group was largely sound, with no deaths or other abnormal behaviors detected. No abnormalities were observed in the ophthalmic examinations of the animals in each group.

#### 3.2.2. Weight, Food Consumption, and Food Efficiency

Compared with the negative control group, female rats in the high‐dose group demonstrated statistically significant increases in body weight during Weeks 11–13 (Figure 1a, *p* < 0.05), while significant reductions in food efficiency were observed in female rats of the low‐dose group at Week 8 and in male rats of the low‐dose group at Week 6 (Figure 2a, *p* < 0.05). No statistically significant differences were detected in body weight, food consumption, food efficiency, total food intake, total weight gain, or overall food efficiency across other dose groups and time points for either sex. Additionally, comparisons between the high‐dose satellite group and the negative control satellite group revealed no significant differences (Figures 1b, 3b, and 2b).

**FIGURE 1:**
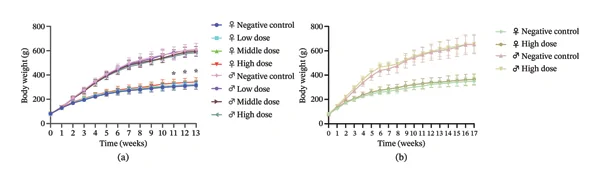
Weekly body weight of animals in the main groups (a) and satellite groups (b). “♀” and “♂” represent females and males, respectively. “^∗^” indicates comparisons between the high‐dose group of female rats and the negative control group (*p* < 0.05).

**FIGURE 2:**
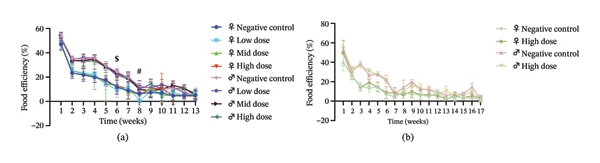
Weekly food efficiency in the main groups (a) and satellite groups (b). “♀” and “♂” represent females and males, respectively. “$” indicates comparisons between the low‐dose group of male rats and the negative control group (*p* < 0.05). “#” indicates comparisons between the low‐dose group of female rats and the negative control group (*p* < 0.05).

**FIGURE 3:**
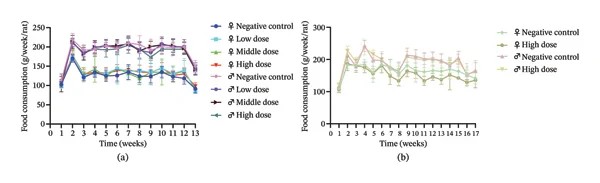
Weekly food consumption in the main groups (a) and satellite groups (b). “♀” and “♂” represent females and males, respectively.

#### 3.2.3. Hematology and Clinical Chemistry

During the mid‐term evaluation, hematological analyses showed no significant differences between the high‐dose satellite group and the negative control satellite group in either female or male rats (Table 2). The clinical chemistry analysis showed that the female rats in the high‐dose satellite group exhibited a significant decrease in the levels of AST, while the male rats in the same group displayed a significant increase in the level of Cl^−^ (Table 2, *p* < 0.05). All other biochemical parameters in both sexes remained comparable to controls.

**TABLE 2:** Hematology and clinical chemistry parameters of satellite groups in rats during mid‐term and recovery phases ($\overset{¯}{x}$ ± SD, *n* = 5).

Parameters	Mid‐term				Recovery phase			
	Female		Male		Female		Male	
	Control	High	Control	High	Control	High	Control	High
WBC (× 10^9^/L)	9.5 ± 1.0	8.3 ± 2.4	9.9 ± 2.1	10.0 ± 1.4	5.7 ± 2.1	4.6 ± 1.5	7.2 ± 1.6	6.7 ± 1.6
LY (%)	77.6 ± 1.5	77.0 ± 4.8	78.5 ± 2.7	70.4 ± 12.4	74.2 ± 3.4	77.8 ± 2.9	73.0 ± 3.0	68.9 ± 7.6
MO (%)	0.5 ± 0.1	0.4 ± 0.2	0.7 ± 0.2	1.3 ± 0.7	0.8 ± 0.2	0.8 ± 0.5	0.9 ± 0.2	1.0 ± 0.4
NEUT (%)	21.2 ± 1.7	21.8 ± 5.0	19.9 ± 2.8	27.5 ± 11.9	23.0 ± 3.5	19.9 ± 2.6	24.2 ± 2.6	28.4 ± 7.0
EO (%)	0.7 ± 0.1	0.9 ± 0.3	0.8 ± 0.1	0.8 ± 0.4	2.0 ± 1.9	1.5 ± 0.6	1.8 ± 0.6	1.7 ± 0.4
BASO (%)	0.0 ± 0.0	0.0 ± 0.0	0.0 ± 0.0	0.0 ± 0.0	0.0 ± 0.0	0.0 ± 0.0	0.0 ± 0.0	0.0 ± 0.0
HGB (g/L)	165 ± 4	160 ± 6	167 ± 3	168 ± 7	142 ± 6	145 ± 4	155 ± 8	153 ± 6
RBC (× 10^12^/L)	8.59 ± 0.34	8.35 ± 0.24	8.61 ± 0.13	8.57 ± 0.48	7.72 ± 0.33	7.75 ± 0.15	9.06 ± 0.61	8.78 ± 0.15
PLT (× 10^3^/μL)	953 ± 97	994 ± 200	997 ± 63	983 ± 109	870 ± 135	960 ± 166	1064 ± 73	1010 ± 97
HCT (%)	45.9 ± 1.3	45.9 ± 1.6	48.3 ± 1.3	48.3 ± 1.3	39.9 ± 1.3	40.9 ± 1.2	43.8 ± 1.7	43.7 ± 1.1
APTT (S)	—	—	—	—	10.1 ± 0.8	9.3 ± 1.3	9.1 ± 1.2	11.8 ± 1.5^∗^
PT (S)	—	—	—	—	10.7 ± 0.2	10.6 ± 0.4	12.4 ± 0.3	13.3 ± 0.9
ALT (U/L)	22.6 ± 4.2	24.6 ± 3.8	33.8 ± 2.8	36.6 ± 6.7	33.0 ± 1.9	36.6 ± 8.7	33.8 ± 6.4	36.8 ± 13.7
AST (U/L)	97.4 ± 8.1	84.6 ± 8.3^∗^	103.2 ± 8.0	117.4 ± 27.9	130.0 ± 15.5	112.6 ± 20.5	112.0 ± 17.1	107.6 ± 11.5
TP (g/L)	69.0 ± 2.1	68.3 ± 5.2	65.9 ± 2.5	64.3 ± 3.5	65.3 ± 3.7	65.2 ± 2.1	61.4 ± 2.5	57.7 ± 3.7
ALB (g/L)	35.9 ± 2.4	35.7 ± 2.9	32.5 ± 1.1	31.6 ± 1.4	36.2 ± 3.2	36.8 ± 2.2	33.1 ± 0.7	31.5 ± 1.7
ALP (U/L)	133.2 ± 15.3	120.0 ± 30.3	180.6 ± 43.8	144.0 ± 19.0	93.8 ± 17.7	75.2 ± 13.7	100.6 ± 23.9	80.0 ± 10.8
GGT (U/L)	28.1 ± 2.7	24.2 ± 4.1	29.1 ± 2.4	26.6 ± 3.7	33.2 ± 2.9	35.1 ± 2.8	34.6 ± 5.1	33.9 ± 5.0
GLU (mmol/L)	6.45 ± 0.80	6.47 ± 0.82	6.10 ± 0.44	6.21 ± 0.60	5.85 ± 0.78	5.90 ± 0.56	6.33 ± 1.66	6.24 ± 0.38
BUN (mmol/L)	3.62 ± 0.27	3.71 ± 0.22	3.84 ± 0.47	3.69 ± 0.76	4.07 ± 0.91	3.51 ± 0.57	3.32 ± 0.30	3.32 ± 0.52
Crea (μmol/L)	50.6 ± 4.1	52.4 ± 1.0	48.8 ± 0.8	48.5 ± 2.2	53.6 ± 3.5	53.3 ± 6.6	46.9 ± 2.0	48.1 ± 8.4
CHO (mmol/L)	1.84 ± 0.37	2.12 ± 0.58	1.69 ± 0.51	2.01 ± 0.54	1.66 ± 0.32	1.81 ± 0.46	1.70 ± 0.26	1.93 ± 0.47
TG (mmol/L)	0.61 ± 0.09	0.66 ± 0.24	0.71 ± 0.29	0.79 ± 0.40	0.67 ± 0.08	0.59 ± 0.10	0.74 ± 0.22	0.78 ± 0.33
K^+^ (mmol/L)	4.85 ± 0.45	4.55 ± 0.24	4.91 ± 0.40	4.53 ± 0.20	4.32 ± 0.27	4.37 ± 0.13	4.56 ± 0.26	4.41 ± 0.21
Na^+^ (mmol/L)	141.0 ± 1.1	141.7 ± 1.2	144.6 ± 0.7	144.7 ± 1.4	138.1 ± 0.9	138.4 ± 1.2	140.5 ± 1.3	140.2 ± 1.3
Cl^−^ (mmol/L)	101.7 ± 0.6	103.3 ± 1.6	101.6 ± 1.3	104.1 ± 1.7^∗^	104.1 ± 2.2	104.8 ± 0.8	105.4 ± 1.2	105.1 ± 1.1

^∗^ *p* < 0.05.

At the terminal phase, hematology analysis showed that the female rats in the high‐dose group showed a significant elevation in the level of APTT compared with controls, while the male rats in all dose groups (low‐, mid‐, and high‐dose groups) exhibited significant decreases in the level of NEUT% and increases in the level of LY%, as well as a significant increase in the level of EO% observed in the high‐dose male group (Table 3, *p* < 0.05). Clinical chemistry analysis revealed a significant reduction in the level of Na^+^ detected in the low‐dose male group, while the level of TG decreased significantly in mid‐ and high‐dose male groups (Table 3, *p* < 0.05). All other parameters in both sexes showed no statistically significant variations.

**TABLE 3:** Hematology and clinical chemistry parameters of main groups in rats during terminal phase ($\overset{¯}{x}$ ± SD, *n* = 10).

Parameters	Female				Male			
	Control	Low	Mid	High	Control	Low	Mid	High
WBC (× 10^9^/L)	3.2 ± 0.9	3.4 ± 0.9	3.1 ± 0.8	3.4 ± 1.0	6.2 ± 1.6	6.7 ± 2.1	5.6 ± 1.5	5.6 ± 1.4
LY (%)	74.9 ± 6.3	78.6 ± 4.0	73.4 ± 4.9	71.9 ± 6.8	70.1 ± 3.3	73.0 ± 4.1^∗^	73.9 ± 2.5^∗^	73.7 ± 2.1^∗^
MO (%)	0.6 ± 0.4	0.3 ± 0.3	0.6 ± 0.6	0.6 ± 0.3	0.9 ± 0.5	0.6 ± 0.2	0.6 ± 0.6	0.6 ± 0.3
NEUT (%)	22.8 ± 5.9	19.6 ± 3.9	24.7 ± 4.3	25.8 ± 6.5	27.9 ± 3.4	25.0 ± 4.0^∗^	24.2 ± 2.7^∗^	24.0 ± 1.9^∗^
EO (%)	1.6 ± 1.0	1.5 ± 0.6	1.4 ± 0.5	1.7 ± 1.1	1.2 ± 0.4	1.5 ± 0.2	1.3 ± 0.6	1.6 ± 0.6^∗^
BASO (%)	0.0 ± 0.0	0.0 ± 0.0	0.0 ± 0.0	0.0 ± 0.0	0.0 ± 0.0	0.0 ± 0.0	0.0 ± 0.0	0.0 ± 0.0
HGB (g/L)	149 ± 6	151 ± 6	150 ± 5	153 ± 6	158 ± 7	158 ± 10	158 ± 6	154 ± 6
RBC (× 10^12^/L)	7.92 ± 0.35	8.29 ± 0.42	8.13 ± 0.50	8.21 ± 0.38	8.93 ± 0.49	8.93 ± 0.55	8.94 ± 0.57	8.79 ± 0.38
PLT (× 10^3^/μL)	928 ± 112	1006 ± 120	1020 ± 129	958 ± 116	1156 ± 101	1177 ± 138	1090 ± 108	1072 ± 76
HCT (%)	42.0 ± 1.5	42.5 ± 1.7	42.6 ± 1.6	43.3 ± 1.7	45.2 ± 1.9	45.0 ± 2.7	44.9 ± 1.4	43.9 ± 1.9
APTT (S)	10.2 ± 1.3	10.1 ± 1.3	10.7 ± 1.2	11.5 ± 1.4^∗^	12.7 ± 1.5	13.0 ± 1.6	11.5 ± 1.7	12.8 ± 1.5
PT (S)	11.0 ± 0.6	11.0 ± 0.6	11.0 ± 0.7	11.1 ± 0.5	13.1 ± 0.7	13.1 ± 1.1	12.8 ± 0.5	13.5 ± 0.9
ALT (U/L)	33 ± 12	28 ± 7	29 ± 6	27 ± 4	32 ± 5	31 ± 6	29 ± 5	32 ± 4
AST (U/L)	130 ± 16	128 ± 26	130 ± 16	129 ± 18	126 ± 16	122 ± 24	117 ± 22	136 ± 14
TP (g/L)	64.0 ± 5.1	64.5 ± 4.0	61.9 ± 3.3	65.1 ± 4.3	62.0 ± 2.9	61.2 ± 1.9	62.6 ± 2.8	61.5 ± 2.5
ALB (g/L)	35.3 ± 3.1	34.7 ± 1.9	33.6 ± 2.1	35.5 ± 2.3	32.2 ± 1.4	31.7 ± 1.0	32.8 ± 1.4	32.5 ± 1.0
ALP (U/L)	103 ± 18	96 ± 24	102 ± 19	104 ± 19	110 ± 16	105 ± 14	112 ± 21	110 ± 10
GGT (U/L)	24.2 ± 6.9	28.0 ± 5.9	23.5 ± 5.7	23.0 ± 4.1	28.3 ± 1.5	26.2 ± 1.0	25.0 ± 0.8	29.5 ± 3.8
GLU (mmol/L)	6.02 ± 0.88	5.67 ± 0.86	6.01 ± 0.57	5.86 ± 0.74	6.22 ± 0.72	5.89 ± 0.99	6.00 ± 1.07	6.21 ± 0.88
BUN (mmol/L)	3.74 ± 1.01	3.56 ± 0.72	3.69 ± 0.47	3.52 ± 0.77	3.43 ± 0.26	3.57 ± 0.47	3.47 ± 0.35	3.25 ± 0.37
Crea (μmol/L)	53.6 ± 7.6	52.4 ± 8.3	52.6 ± 4.2	49.5 ± 5.2	51.2 ± 4.2	52.4 ± 4.3	52.8 ± 3.9	51.6 ± 3.6
CHO (mmol/L)	1.39 ± 0.25	1.47 ± 0.28	1.47 ± 0.28	1.38 ± 0.26	1.45 ± 0.40	1.20 ± 0.21	1.41 ± 0.34	1.44 ± 0.24
TG (mmol/L)	0.61 ± 0.12	0.64 ± 0.10	0.69 ± 0.12	0.62 ± 0.12	0.99 ± 0.43	0.92 ± 0.58	0.62 ± 0.25^∗^	0.60 ± 0.27^∗^
K^+^ (mmol/L)	4.33 ± 0.42	4.36 ± 0.32	4.37 ± 0.19	4.38 ± 0.31	4.65 ± 0.26	4.71 ± 0.24	4.65 ± 0.29	4.65 ± 0.12
Na^+^ (mmol/L)	141.0 ± 1.1	141.4 ± 1.1	141.3 ± 0.9	141.0 ± 1.2	143.0 ± 0.8	142.0 ± 1.1^∗^	142.8 ± 1.2	143.3 ± 0.9
Cl^−^ (mmol/L)	106.6 ± 1.5	106.5 ± 1.2	106.7 ± 1.0	106.5 ± 1.6	106.7 ± 1.0	106.3 ± 1.2	106.7 ± 1.5	107.1 ± 1.2

^∗^ *p* < 0.05.

During the recovery phase, a significant increase was detected in the level of APTT in male rats of the high‐dose satellite group (Table 2, *p* < 0.05), while no statistically significant differences were observed in other hematological or biochemical parameters between the high‐dose satellite group and the negative control satellite group for either sex (Table 2).

#### 3.2.4. Urine Analysis

Compared with the negative control group, no significant differences were observed in all the tested indicators for either female or male rats during the mid‐term and recovery phases of the trial (Table 4). At the terminal phase, the elevated SG values were detected in male rats of the high‐dose group and an increased KET positivity rate was revealed in male rats of the low‐dose group, whereas no significant differences were observed in other test indicators for female or male rats across the other dose groups (Table 5).

**TABLE 4:** Urine results of satellite groups in rats during mid‐term and recovery phases (*n* = 5).

Item	Degree	Mid‐term				Recovery phase			
		Female		Male		Female		Male	
		Control	High	Control	High	Control	High	Control	High
pH	6.5	0	0	0	0	0	1	0	0
	7.0	1	1	3	0	0	1	2	1
	7.5	3	3	2	4	3	3	2	2
	8.0	0	1	0	1	2	0	1	2
	8.5	1	0	0	0	0	0	0	0

GLU	−	5	5	5	5	5	5	5	5

PRO	−	4	4	4	5	4	5	4	4
	+ −	1	1	1	0	1	0	1	1

BLD	−	5	5	5	4	5	5	4	4
	1 +	0	0	0	1	0	0	0	0
	2 +	0	0	0	0	0	0	1	0
	3 +	0	0	0	0	0	0	0	1

SG	1.010	0	1	1	0	0	0	1	0
	1.015	3	2	2	5	4	4	3	3
	1.020	2	2	1	0	1	1	1	2
	1.025	0	0	1	0	0	0	0	0

BIL	−	5	5	5	5	5	5	5	5

UBG	Normal 3.4	5	5	4	5	5	5	4	4
	Normal 17	0	0	1	0	0	0	1	1

KET	−	5	5	0	0	5	5	0	0
	+ −	0	0	4	5	0	0	5	4
	1 +	0	0	1	0	0	0	0	1

**TABLE 5:** Urine results of main groups in rats during terminal phase (*n* = 10).

Item	Degree	Female				Male			
		Control	Low	Mid	High	Control	Low	Mid	High
pH	6.5	0	0	0	0	0	0	0	0
	7.0	2	1	2	3	0	0	0	0
	7.5	3	4	4	4	8	5	6	7
	8.0	4	2	2	1	1	2	2	2
	8.5	1	3	2	2	1	3	2	1

GLU	−	10	10	10	10	10	10	10	10

PRO	−	8	8	7	8	8	9	9	8
	+ −	1	1	3	2	2	1	0	2
	1 +	1	0	0	0	0	0	1	0
	2 +	0	1	0	0	0	0	0	0

BLD	−	10	10	10	10	10	10	10	10

SG	1.010	0	0	1	1	1	2	2	0
	1.015	6	7	4	3	9	6	7	7
	1.020	3	3	4	4	0	2	1	3^∗^
	1.025	0	0	1	2	0	0	0	0
	≥ 1.030	1	0	0	0	0	0	0	0

BIL	−	10	10	7	10	10	10	10	10
	1 +	0	0	3	0	0	0	0	0

UBG	Normal 3.4	10	10	7	10	10	10	10	10
	Normal 17	0	0	3	0	0	0	0	0

KET	−	9	10	10	10	4	0	4	1
	+ –	1	0	0	0	6	8	6	7
	1 +	0	0	0	0	0	2^∗^	0	2

^∗^ *p* < 0.05.

#### 3.2.5. Histology and Pathology

Compared with the negative control group, no significant differences were observed in the absolute organ weights or organ coefficient of either female or male rats in all dose groups at the terminal phase (Table 6) or the recovery phase (Table 7).

**TABLE 6:** Absolute organ weights and organ coefficient of main groups in rats during terminal phase ($\overset{¯}{x}$ ± SD, *n* = 10).

Organ	Female				Male			
	Control	Low	Mid	High	Control	Low	Mid	High
Body weight (g)	293.9 ± 32.0	304.1 ± 22.1	302.0 ± 28.8	319.3 ± 34.3	580.9 ± 51.7	577.4 ± 52.7	560.6 ± 44.6	550.9 ± 28.0
Brain (g)	1.86 ± 0.12	1.87 ± 0.14	1.93 ± 0.09	1.95 ± 0.14	2.16 ± 0.12	2.13 ± 0.10	2.10 ± 0.12	2.09 ± 0.12
Relative weight (%)	0.64 ± 0.06	0.62 ± 0.07	0.64 ± 0.07	0.61 ± 0.06	0.37 ± 0.04	0.37 ± 0.04	0.38 ± 0.03	0.38 ± 0.03
Heart (g)	0.93 ± 0.09	0.90 ± 0.04	0.99 ± 0.15	1.01 ± 0.16	1.71 ± 0.14	1.68 ± 0.07	1.69 ± 0.24	1.63 ± 0.21
Relative weight (%)	0.32 ± 0.04	0.30 ± 0.03	0.33 ± 0.03	0.32 ± 0.05	0.30 ± 0.03	0.29 ± 0.03	0.30 ± 0.04	0.30 ± 0.04
Liver (g)	6.42 ± 0.81	6.70 ± 0.67	6.99 ± 0.90	6.89 ± 0.75	14.13 ± 1.63	13.34 ± 2.11	12.85 ± 1.46	13.33 ± 0.46
Relative weight (%)	2.19 ± 0.26	2.20 ± 0.15	2.31 ± 0.14	2.18 ± 0.30	2.44 ± 0.28	2.30 ± 0.20	2.29 ± 0.19	2.42 ± 0.10
Spleen (g)	0.46 ± 0.08	0.51 ± 0.06	0.52 ± 0.07	0.51 ± 0.12	0.95 ± 0.14	0.93 ± 0.17	0.82 ± 0.15	0.82 ± 0.15
Relative weight (%)	0.16 ± 0.03	0.17 ± 0.02	0.17 ± 0.02	0.16 ± 0.03	0.16 ± 0.02	0.16 ± 0.02	0.15 ± 0.02	0.15 ± 0.03
Kidney (g)	1.65 ± 0.18	1.76 ± 0.27	1.74 ± 0.18	1.79 ± 0.17	3.18 ± 0.27	3.18 ± 0.25	3.02 ± 0.38	3.09 ± 0.37
Relative weight (%)	0.56 ± 0.07	0.58 ± 0.07	0.58 ± 0.04	0.57 ± 0.08	0.55 ± 0.06	0.55 ± 0.04	0.54 ± 0.04	0.56 ± 0.06
Uterus (g)	0.51 ± 0.10	0.60 ± 0.11	0.59 ± 0.16	0.62 ± 0.17	—	—	—	—
Relative weight (%)	0.18 ± 0.04	0.20 ± 0.05	0.19 ± 0.05	0.20 ± 0.05	—	—	—	—
Ovaries (g)	0.164 ± 0.031	0.170 ± 0.046	0.169 ± 0.048	0.164 ± 0.028	—	—	—	—
Relative weight (%)	0.057 ± 0.013	0.056 ± 0.016	0.056 ± 0.017	0.052 ± 0.014	—	—	—	—
Epididymides (g)	—	—	—	—	1.61 ± 0.12	1.70 ± 0.28	1.59 ± 0.31	1.62 ± 0.16
Relative weight (%)	—	—	—	—	0.28 ± 0.03	0.30 ± 0.05	0.28 ± 0.05	0.29 ± 0.03
Testis (g)	—	—	—	—	3.77 ± 0.35	3.79 ± 0.32	3.73 ± 0.32	3.56 ± 0.20
Relative weight (%)	—	—	—	—	0.65 ± 0.08	0.66 ± 0.08	0.67 ± 0.07	0.65 ± 0.03
Thymus (g)	0.326 ± 0.143	0.389 ± 0.085	0.380 ± 0.075	0.308 ± 0.081	0.544 ± 0.099	0.576 ± 0.137	0.484 ± 0.066	0.528 ± 0.137
Relative weight (%)	0.111 ± 0.046	0.128 ± 0.024	0.125 ± 0.019	0.096 ± 0.024	0.093 ± 0.014	0.100 ± 0.022	0.086 ± 0.008	0.096 ± 0.024
Adrenal gland (g)	0.070 ± 0.015	0.067 ± 0.021	0.066 ± 0.015	0.072 ± 0.010	0.071 ± 0.021	0.072 ± 0.021	0.104 ± 0.131	0.063 ± 0.020
Relative weight (%)	0.024 ± 0.005	0.022 ± 0.006	0.022 ± 0.005	0.023 ± 0.004	0.012 ± 0.003	0.013 ± 0.005	0.019 ± 0.023	0.011 ± 0.004

**TABLE 7:** Absolute organ weights and organ coefficient of satellite groups in rats during recovery phase ($\overset{¯}{x}$ ± SD, *n* = 5).

Organ	Female		Male	
	Control	High	Control	High
Body weight (g)	332.0 ± 33.1	348.9 ± 45.3	624.6 ± 72.8	622.6 ± 68.7
Brain (g)	1.95 ± 0.15	2.03 ± 0.08	2.16 ± 0.07	2.21 ± 0.05
Relative weight (%)	0.59 ± 0.04	0.59 ± 0.06	0.35 ± 0.04	0.36 ± 0.04
Heart (g)	1.01 ± 0.06	1.01 ± 0.12	1.64 ± 0.37	1.66 ± 0.23
Relative weight (%)	0.30 ± 0.02	0.29 ± 0.03	0.26 ± 0.05	0.27 ± 0.01
Liver (g)	7.40 ± 0.67	7.76 ± 1.23	14.23 ± 2.55	14.55 ± 2.40
Relative weight (%)	2.23 ± 0.10	2.22 ± 0.09	2.27 ± 0.18	2.33 ± 0.25
Spleen (g)	0.54 ± 0.12	0.56 ± 0.09	0.86 ± 0.13	0.88 ± 0.18
Relative weight (%)	0.16 ± 0.03	0.16 ± 0.01	0.14 ± 0.01	0.14 ± 0.02
Kidney (g)	1.86 ± 0.21	1.97 ± 0.35	2.94 ± 0.27	3.07 ± 0.80
Relative weight (%)	0.56 ± 0.03	0.57 ± 0.07	0.47 ± 0.03	0.49 ± 0.08
Uterus (g)	0.78 ± 0.30	0.69 ± 0.24	—	—
Relative weight (%)	0.23 ± 0.09	0.20 ± 0.08	—	—
Ovaries (g)	0.162 ± 0.034	0.189 ± 0.014	—	—
Relative weight (%)	0.049 ± 0.012	0.055 ± 0.007	—	—
Epididymides (g)	—	—	2.06 ± 0.57	1.70 ± 0.32
Relative weight (%)	—	—	0.33 ± 0.06	0.27 ± 0.03
Testis (g)	—	—	3.76 ± 0.46	4.02 ± 0.68
Relative weight (%)	—	—	0.60 ± 0.03	0.64 ± 0.07
Thymus (g)	0.391 ± 0.078	0.505 ± 0.179	0.460 ± 0.064	0.476 ± 0.174
Relative weight (%)	0.118 ± 0.021	0.144 ± 0.043	0.074 ± 0.010	0.075 ± 0.024
Adrenal gland (g)	0.068 ± 0.017	0.152 ± 0.183	0.082 ± 0.043	0.073 ± 0.021
Relative weight (%)	0.020 ± 0.004	0.046 ± 0.059	0.013 ± 0.006	0.012 ± 0.004

The high‐dose group and the control group exhibited lesions primarily characterized by a group of characteristic lesions, such as inflammatory cell infiltration in the liver, alveolar foam cell aggregation, increased glandular tubule‐like structure in the thymus, inflammatory cell infiltration within myocardial fibers, pulmonary ossification, and hyaline casts in renal tubules and inflammatory cell infiltration in the stroma of the prostate. However, the aforementioned lesions were observed in both the control group and the high‐dose group, with no statistically significant difference (Table 8).

**TABLE 8:** Histological results of satellite groups in rats during terminal and recovery phases.

Lesion	Terminal phase (*n* = 10)				Recovery phase (*n* = 5)			
	Female		Male		Female		Male	
	Control	High	Control	High	Control	High	Control	High
Alveolar foam cell aggregation	1	1	0	2	0	0	1	0
Gastric gland ectasia	0	1	2	0	0	1	0	0
Inflammatory cell infiltration in liver	1	1	1	1	0	1	0	0
Mild steatosis in liver	0	1	0	0	1	0	0	0
Inflammatory cell infiltrate in stroma of renal	1	0	0	0	0	0	0	0
Increased glandular tubule‐like structure in thymus	0	1	2	0	0	0	0	0
Mild inflammatory cell infiltration within myocardial fibers	0	0	2	2	0	0	0	0
Inflammatory cell infiltration within myocardial fibers	0	0	0	0	0	0	0	1
Pulmonary ossification	0	0	1	0	0	0	0	0
Pancreatic atrophy	0	0	1	0	0	0	0	0
Hyaline casts in renal tubules	0	0	1	0	0	0	0	0
Inflammatory cell infiltration in the stroma of the prostate	0	0	2	1	0	0	1	0
Uterine cavity ectasia	0	0	0	0	1	0	0	0

### 3.3. Teratogenic Toxicity in Pregnant Rats

#### 3.3.1. General Condition and Weight of Pregnant Rats

During the trial, the pregnant rats in all groups exhibited good general condition, with no obvious abnormal symptoms or signs observed. Compared with the negative control group, no significant differences (*p* > 0.05) were observed in body weight, weight gain, or net weight gain among the different dose groups (Table 9).

**TABLE 9:** Body weight of pregnant rats.

Group	N	Day 0	Day 6	Day 9	Day 12	Day 15	Day 20	Weight gain (g)	Net weight gain (g)
Negative	16	260.0 ± 43.6	295.0 ± 38.8	305.9 ± 37.2	323.2 ± 37.2	343.0 ± 35.8	410.8 ± 37.5	115.8 ± 17.8	37.3 ± 10.1
Low	16	266.7 ± 46.2	306.2 ± 40.4	315.7 ± 38.4	337.0 ± 36.7	354.0 ± 36.2	421.2 ± 47.0	115.0 ± 27.4	36.9 ± 18.7
Mid	16	263.2 ± 35.6	305.2 ± 29.6	314.5 ± 25.5	330.7 ± 24.1	346.2 ± 22.1	412.8 ± 26.7	107.7 ± 35.2	42.9 ± 25.9
High	16	274.9 ± 44.1	305.5 ± 43.0	318.6 ± 40.6	335.6 ± 41.9	349.4 ± 41.1	410.6 ± 46.3	105.1 ± 18.4	36.7 ± 24.5
Positive	18	319.5 ± 22.1	348.8 ± 24.1	363.3 ± 25.6	378.1 ± 28.0	387.8 ± 30.7	424.6 ± 40.8	75.8 ± 23.4	30.7 ± 16.6

*Note:* Weight gain = maternal body weight at dissection − gestational body weight on Day 6. Net weight gain = maternal weight at dissection − weight of uterus in conjunction with fetus − gestational body weight on Day 6.

#### 3.3.2. Embryonic Formation in Pregnant Rats

Compared with the negative control group, the absorbed fetus rate was significantly increased in the low‐ and mid‐dose groups (*p* < 0.01). However, no significant differences were observed in the number of corpora lutea, implantation, viable/nonviable fetus, or the nonviable fetus rate among all the dose groups (Table 10).

**TABLE 10:** Embryonic formation in pregnant rats.

Group	Corpus luteum (*n*)	Implantation (*n*)	Viable fetus (*n*)	Female/male (*n*/*n*)	Nonviable fetus (*n*)	Absorbed fetus (*n*)	Nonviable fetus rate (%)	Absorbed fetus rate (%)
Negative	279	206	203	98/105	0	3	0.00	1.46
Low	279	218	201	103/98	0	17	0.00	7.80^∗∗^
Mid	281	190	174	77/97	0	16	0.00	8.42^∗∗^
High	254	174	170	89/81	0	4	0.00	2.30
Positive	330	243	210	132/78	2	31	0.94	12.76

^∗∗^ *p* < 0.01.

#### 3.3.3. Appearance and Development of Fetal Rats

Compared with the negative control group, no significant differences were observed in the uterine weight with fetuses, fetal body weight, body length, external malformation rate, and the litter rate of external malformations among the various dose groups (Table 11).

**TABLE 11:** Body weight, body length, and external malformations of fetal rats.

Group	Fetal rat (*n*)	Uterine weight with fetus (g)	Body weight (g)	Body length (mm)	External malformation (*n*)					Litter rate (%)
					Encephalocele	Tailless	No toe	Total	Malformation rate (%)	
Negative	203	78.4 ± 15.5	4.05 ± 0.68	60.60 ± 4.04	0	0	0	0	0.0	0/0.0
Low	201	78.1 ± 25.4	4.03 ± 0.60	58.95 ± 4.44	0	0	0	0	0.0	0/0.0
Mid	174	64.8 ± 25.6	3.84 ± 0.44	58.99 ± 2.93	0	0	0	0	0.00	0/0.00
High	170	68.4 ± 25.1	4.17 ± 0.48	61.74 ± 3.96	0	0	0	0	0.0	0/0.0
Positive	210	45.0 ± 18.5	2.12 ± 0.29	37.31 ± 4.21	210	50	0	210	100	17/100

#### 3.3.4. Visceral and Skeletal Malformation of Fetal Rats

There was no significant difference in the number of visceral abnormalities and the litter rate of visceral abnormalities among the fetal rats in each dose group, and the visceral malformation of individual fetal rats was mainly manifested as hydronephrosis (Table 12).

**TABLE 12:** Viscera abnormalities of fetal rats.

Group	Fetal rat (*n*)	Visceral malformations or abnormalities (*n*)						Litter rate (%)
		Brain	Eye	Palate	Hydronephrosis	Total	Malformation rate (%)	
Negative	98	0	0	0	5	5	5.10	2/12.5
Low	95	0	0	0	5	5	5.26	3/18.8
Mid	84	0	0	0	7	7	8.33	3/18.8
High	80	0	0	0	7	7	8.75	3/18.8
Positive	100	69	1	0	18	69	69.0	16/100.0

There was no significant difference in the rate of skeletal abnormality and the litter rate of skeletal abnormality among the fetal rats in each dose group, and the skeletal abnormality of individual fetal rats was mainly manifested as absence of the 5th and 6th sternum or ossification insufficiency (Table 13).

**TABLE 13:** Skeletal abnormalities of fetal rats.

Group	Fetal rat (*n*)	Skeletal malformations or abnormalities (*n*)							Litter rate (%)
		Skull	Sternum	Ribs	Vertebra	Limb bones	Total	Malformation rate (%)	
Negative	105	0	1	0	0	0	1	0.95	1/6.3
Low	106	0	3	0	0	0	3	2.83	3/18.8
Mid	90	0	3	0	0	0	3	3.33	2/12.5
High	90	0	1	0	0	0	1	1.11	1/6.3
Positive	110	110	93	107	0	0	110	100.0	17/100.0

### 3.4. Genotoxicity

#### 3.4.1. Mammalian Erythrocyte Micronucleus Test

Compared with the negative control group, the positive control group showed a statistically significant difference in micronucleus frequency of both sexes. In contrast, no significant differences in micronucleus frequency were observed in all dose groups of the test substance (Table 14). The proportion of PCEs was no less than 20% of that in the negative control group.

**TABLE 14:** Mammalian erythrocyte micronucleus in mice.

Gender	Dose (g/kg BW)	Number of animals (PCS)	PCE (PCS)	NCE (PCS)	PCE (%)	Micronucleus		
						Observed PCE (PCS)	PCE	Micronucleus frequency (‰)
Female	0	5	1000	827	54.7	10,000	34	3.4 ± 2.3
	2.5	5	1000	847	54.1	10,000	44	4.4 ± 2.5
	5.0	5	1000	871	53.4	10,000	42	4.2 ± 2.4
	10.0	5	1000	883	53.1	10,000	32	3.2 ± 2.9
	0.04 (CP)	5	1000	894	52.8	10,000	210	21.0 ± 6.5^∗∗^

Male	0	5	1000	828	54.7	10,000	42	4.2 ± 2.4
	2.5	5	1000	870	53.5	10,000	32	3.2 ± 2.4
	5.0	5	1000	870	53.5	10,000	54	5.4 ± 0.5
	10.0	5	1000	874	53.4	10,000	42	4.2 ± 2.4
	0.04 (CP)	5	1000	896	52.7	10,000	200	20.0 ± 5.0^∗∗^

*Note:* PCE, polychromatic erythrocytes; NCE, normochromatic erythrocytes.

^∗∗^ *p* < 0.01.

#### 3.4.2. Chromosome Aberration

Compared with the negative control group, the positive control group (+S9/−S9) exhibited a statistically significant increase in the frequency of chromosomal aberrations in cells (*p* < 0.01). In contrast, all dose groups of the test substance (+S9/−S9) showed no statistically significant differences (Table 15).

**TABLE 15:** Chromosome aberration of mammalian cells in vitro.

Group (4h)		Dose (μg/mL)	Cells observed (%)		Number of cells with chromosomal aberration^#^		Aberration rate^1^ (%)	
			+S9	−S9	+S9	−S9	+S9	−S9
Negative	Distilled water	0	200	200	7 (9.1)	7 (7.0)	3.5	3.5

Positive	CP	15.0	200	—	45 (10.2)	—	22.5^∗∗^	—
	MMC	0.8	—	200	—	48 (12.3)	—	24.0^∗∗^

SOD		5000	200	200	5 (8.0)	6 (5.1)	2.5	3.0
		2000	200	200	5 (6.2)	5 (6.0)	2.5	2.5
		800	200	200	5 (8.0)	5 (8.0)	2.5	2.5

**Group (24h)**		**Dose (μg/mL)**	**Cells observed (%)**		Number of cells with chromosomal aberration **^#^**		**Aberration rate** ^1^ **(%)**	
			**−S9**		**−S9**		**−S9**	

Negative	Distilled water	0	200		7 (8.2)		3.5	

Positive	MMC	0.8	200		40 (11.0)		20.0^∗∗^	

SOD		5000	200		6 (9.1)		3.0	
		2000	200		6 (5.0)		3.0	
		800	200		5 (6.1)		2.5	

^∗∗^ *p* < 0.01, based on comparison with negative control group.

^#^Number of cells with chromosomal structural aberrations (chromosomal gaps and numerical aberrations).

^1^Aberration rate statistics with exclusion of chromosomal gaps and numerical aberrations.

#### 3.4.3. Revertant Mutations in *Salmonella typhimurium*

SOD showed no significant antibacterial effect toward all five tested *S. typhimurium* strains (TA97a, TA98, TA100, TA102, and TA1535) with or without S9 activation. The number of revertant colonies in each dose group did not exceed twice that of the spontaneous revertant group, and there was no dose–response relationship or reproducible positive result. Meanwhile, SOD remained completely soluble in distilled water at all tested concentrations (up to 5000 μg/plate), and no precipitate was observed in the upper agar layer. However, the positive controls demonstrated mutagenic effects on the corresponding strains used in this assay (Table 16, Supporting Table S1).

**TABLE 16:** Reverse mutation assay.

Group		SOD					Negative control	Spontaneous mutation	Positive control	
Dose (μg/plate)		128	320	800	2000	5000	0	0	Substance (μg/plate)	
TA97a	−S9	95 ± 9 (1.0)	95 ± 11 (1.0)	104 ± 13 (1.1)	100 ± 8 (1.1)	93 ± 5 (1.0)	91 ± 8 (1.0)	95 ± 11	Fenaminosulf (50.0)	897 ± 71 (9.4)
	+S9	88 ± 6 (0.9)	97 ± 3 (1.0)	103 ± 13 (1.0)	99 ± 13 (1.0)	88 ± 9 (0.9)	88 ± 4 (0.9)	99 ± 10	2‐Aminofluorene (10.0)	843 ± 82 (8.5)

TA98	−S9	31 ± 6 (1.0)	30 ± 4 (1.0)	29 ± 3 (1.0)	30 ± 3 (1.0)	30 ± 3 (1.0)	34 ± 2 (1.1)	30 ± 4	Fenaminosulf (50.0)	812 ± 42 (27.1)
	+S9	32 ± 5 (1.0)	31 ± 3 (0.9)	33 ± 3 (1.0)	33 ± 5 (1.0)	32 ± 6 (1.0)	30 ± 3 (0.9)	33 ± 5	2‐Aminofluorene (10.0)	923 ± 70 (28.0)

TA100	−S9	218 ± 24 (1.0)	215 ± 6 (1.0)	223 ± 16 (1.0)	227 ± 27 (1.0)	226 ± 22 (1.0)	241 ± 16 (1.1)	225 ± 24	Sodium azide (2.0)	849 ± 79 (3.8)
	+S9	222 ± 37 (0.9)	243 ± 8 (1.0)	244 ± 5 (1.0)	241 ± 13 (1.0)	238 ± 5 (1.0)	225 ± 30 (0.9)	240 ± 16	2‐Aminofluorene (10.0)	985 ± 37 (4.1)

TA102	−S9	359 ± 20 (1.0)	363 ± 11 (1.0)	367 ± 16 (1.0)	358 ± 16 (1.0)	367 ± 14 (1.0)	366 ± 15 (1.0)	359 ± 19	Fenaminosulf (50.0)	951 ± 60 (2.7)
	+S9	355 ± 7 (1.0)	359 ± 25 (1.0)	348 ± 20 (1.0)	356 ± 11 (1.0)	356 ± 17 (1.0)	309 ± 17 (0.9)	351 ± 19	1,8‐Dihydroxyanthraquinone (50.0)	977 ± 173 (2.8)

TA1535	−S9	13 ± 5 (0.8)	12 ± 2 (0.8)	15 ± 4 (1.0)	12 ± 2 (0.8)	12 ± 2 (0.8)	8 ± 2 (0.6)	15 ± 1	Sodium azide (2.0)	504 ± 74 (33.6)
	+S9	14 ± 3 (1.1)	13 ± 2 (1.0)	14 ± 2 (1.1)	13 ± 2 (1.0)	13 ± 2 (1.0)	10 ± 3 (0.7)	13 ± 3	Cyclophosphamide monohydrate (100.0)	440 ± 22 (33.0)

## 4. Discussion and Conclusion

To date, there is no globally unified standard for the daily intake of SOD for human populations. In the European Union, SOD is classified as a food enzyme, which must comply with Regulation (EC) No. 1332/2008 [36], requiring evaluation by the European Food Safety Authority (EFSA) for inclusion in the permitted list. However, SOD has not been approved as a food enzyme as of 2024. When marketed as a dietary supplement, SOD falls under Regulation (EC) No. 1924/2006 and requires substantiation of safety [37], and commercially available products typically provide doses of 50–200 U/day (e.g., MelonSOD, Bionov). In the United States, the regulatory pathway involves Generally Recognized As Safe (GRAS) certification. However, no SOD product (regardless of source) has currently obtained GRAS status. It is regulated under the Dietary Supplement Health and Education Act (DSHEA) of 1994 as a dietary supplement and must comply with Good Manufacturing Practices (GMP), with a typical dose range of 100–400 U/day (e.g., SOD Booster, Life Extension). In China, SOD was historically approved for use in health food products at doses up to 4000 U/day. However, under the updated regulatory framework implemented post‐2021, SOD is now required to undergo safety evaluation and registration as a novel food ingredient [23]. To date, available public records indicate no officially approved cases. SOD, a pivotal endogenous antioxidant enzyme, plays an indispensable role in mitigating oxidative stress and protecting biomacromolecules from ROS‐induced damage. Its broad applications span medicine, agriculture, cosmetics, and food industries, underscoring the necessity for a rigorous safety assessment to support its use as a novel food ingredient.

In contrast to the toxicological assessments by Carillon et al. using a multicomponent, SOD‐rich melon (not GMO) concentrate, our study employed high‐purity SOD lyophilized powder without other antioxidant constituents. The evaluation results thus provide a more accurate assessment of the intrinsic safety profile of SOD [21, 38]. In the acute oral toxicity study, SOD demonstrated no mortality or adverse clinical characteristics at the maximum feasible dose of 10,000 mg/kg BW in mice, supporting its classification as “practically nontoxic.” This aligns with existing literature on SOD safety and establishes a high margin of safety for acute exposure. In the subchronic toxicity study, the statistically significant increase in body weight observed in high‐dose female rats during Weeks 11–13, while reaching statistical significance, is unlikely to be biologically meaningful or treatment‐related for several reasons. First, this effect was transient (limited to Weeks 11–13) and did not persist throughout the 90‐day study period. Second, it was not accompanied by significant changes in food consumption or food efficiency during the same period, nor by changes in cumulative weight gain. Only in the low‐dose group, a significant decrease in food utilization efficiency was observed in female rats at Week 8 and in male rats at Week 6. Third, no similar effects were observed in male rats or in other dose groups. Fourth, this isolated finding lacks a dose–response relationship. Collectively, these observations indicate that the transient body weight change represents a sporadic statistical variation rather than a true treatment‐related biological effect.

At the same time, several hematological, biochemical, and urinary parameters showed statistically significant differences between treated and control groups in this study, including elevated chloride (Cl^−^) in high‐dose males (mid‐term), increased APTT in high‐dose males (terminal), decreased NEUT%, and increased LY% in males across all dose groups, reduced TG in mid‐ and high‐dose males, decreased sodium (Na^+^) in low‐dose males, as well as changes in urine SG and KET positivity in some male groups. However, these findings are not considered toxicologically relevant for the following reasons: (i) They lacked a dose–response relationship; (ii) all values remained within historical control ranges for Wistar rats; (iii) most changes were reversible during the recovery period; (iv) no corresponding histopathological lesions were found in the relevant organs. Furthermore, no treatment‐related clinical signs were observed. Therefore, these sporadic statistical differences are interpreted as incidental biological variation rather than adverse effects of SOD administration. An integrated analysis of biochemical and histopathological findings further supports the safety profile of SOD. Serum biomarkers of liver function (ALT, AST, ALP, GGT) and kidney function (BUN, Crea) showed no treatment‐related alterations across any dose group (Table 3). Consistent with these biochemical findings, histopathological examination revealed no substance‐specific lesions in the liver or kidneys of treated animals (Table 8). Collectively, these findings support a No Observed Adverse Effect Level (NOAEL) of 75 mg/kg BW/day for both sexes in subchronic exposure.

Teratogenicity studies indicated no adverse effects on maternal health, fetal viability, or structural development (external, visceral, or skeletal) at all tested doses. Although increased absorbed fetus rates occurred in low‐ and mid‐dose groups (7.80%–8.42% vs. 1.46% in controls), the absence of a dose–response relationship, the consistency of the results were consistent with the background range reported by Wistar rats in historical control data (typically 5%–10%), and the lack of corroborating developmental toxicity suggested that this finding lacks biological significance. The NOAEL for maternal and developmental toxicity was thus established at 75 mg/kg BW/day.

It should also be noted that the NOAEL of 75 mg/kg BW/day corresponds to the highest dose tested in the 90‐day subchronic study. While this establishes that no adverse effects were observed at this dose level under the study conditions, the true upper limit of safety was not determined. Nevertheless, this NOAEL provides a substantial safety margin relative to the anticipated human intake levels, which are estimated to be in the range of 0.0833–0.667 mg/kg BW/day (based on 50–400 U/day for commercially available SOD supplements). The approximately 100‐ to 900‐fold safety margin further supports the safe use of SOD as a novel food ingredient. Future studies with higher dose levels or longer exposure durations may help establish the true upper limit of safety.

A further limitation concerns the 90‐day study duration, which is substantially shorter than the observation periods required for phenotype onset in transgenic SOD1 models, such as the 115–192 days reported by Jaarsma et al. [39]. Although oral administration limits systemic exposure to intact protein [40], age‐related declines in proteostasis [41], the conformational plasticity of SOD1 [42, 43], and naturally occurring variants [44] could theoretically influence long‐term responses. These considerations temper the interpretation that exogenous SOD1 is necessarily innocuous based solely on the absence of phenotypes in healthy animals over the duration examined. Longer‐term studies in aged animals and with consideration of genetic variation may therefore be warranted.

All three genotoxicity assays yielded negative results. SOD induced no chromosomal damage in vitro or mutagenicity in *Salmonella* strains, even at concentrations up to 5000 μg/mL (chromosome assay) or 5000 μg/plate (Ames test). The micronucleus assay further confirmed no clastogenic/aneugenic effects in vivo at 10,000 mg/kg BW. These outcomes robustly demonstrate the absence of genotoxic risk caused by SOD.

In conclusion, this integrated safety assessment confirms that SOD, at doses substantially exceeding anticipated human intake levels (0.0833 mg/kg BW/day), exhibits no evidence of systemic, developmental, or genetic toxicity. Isolated subclinical alterations observed in subchronic studies were transient, toxicologically insignificant, and reversible. These data, generated under Good Laboratory Practice (GLP) compliant and internationally harmonized test conditions, provide a robust scientific foundation for the safe use of SOD as a novel food ingredient in China and globally. Nevertheless, the findings should be interpreted within the context of the study design, and future research may explore long‐term chronic toxicity, aged populations, and genetic variability to further validate and extend these findings.

## Author Contributions

Disi Chen: writing–original draft and investigation. Xianghong Ke: writing–methodology and investigation. Xiaoya Du, Qiwen Huang, Hongxin Peng, Qing Yu, Jie Tian, Jingjing Qu, Ying Xia, and Yingjing Zhang: investigation. Wei Zhou and Bolin Fan: supervision and methodology.

## Funding

The study was supported by the National Key Research and Development Program of China, 2023YFF1104804, and the Hubei Provincial Public Health Academic Leader Program, 2021–73.

## Conflicts of Interest

The authors declare no conflicts of interest.

## Supporting Information

Additional supporting information can be found online in the Supporting Information section.

## Supporting information


**Supporting Information** Supporting Table S1: Bacterial reverse mutation assay, with only dose variation.

## Data Availability

The data that support the findings of this study are available from the corresponding author upon reasonable request.
