# Supplementary material for: A Comprehensive Toxicological Safety Assessment of Superoxide Dismutase as a Novel Food Ingredient
Source: J Toxicol. 2026 Oct 29;2026:5535992. doi: 10.1155/jt/5535992 (PMC13717387; doi:10.1155/jt/5535992)
Supplement: Supplementary file 1 [file JT-2026-5535992-s001.docx]

**Supplementary Table S1**

| Group | | SOD | | | | | Negative control | Spontaneous mutation | Positive control | |
| --- | --- | --- | --- | --- | --- | --- | --- | --- | --- | --- |
|  | |  | | | | |  |  |  | |
| Dose (μg/plate) | | 8 | 40 | 200 | 1000 | 5000 | 0 | 0 | Substance (μg/plate) |  |
| TA97a | –S9 | 87±11  (0.9) | 97±10  (1.0) | 92±11  (0.9) | 101±10  (1.0) | 101±12  (1.0) | 104±12  (1.1) | 97±3 | Fenaminosulf (50.0) | 824±49  (8.5) |
|  | +S9 | 98±13  (1.0) | 106±11  (1.1) | 101±10  (1.1) | 93±10  (1.0) | 98±6  (1.1) | 98±15  (1.0) | 93±7 | 2-aminofluorene (10.0) | 837±79  (9.0) |
| TA98 | –S9 | 36±2  (1.1) | 38±6  (1.2) | 38±2  (1.2) | 34±4  (1.1) | 37±5  (1.2) | 31±3  (1.0) | 31±3 | Fenaminosulf (50.0) | 955±161  (30.5) |
|  | +S9 | 41±4  (1.0) | 41±2  (1.0) | 43±3  (1.1) | 41±3  (1.0) | 42±1  (1.1) | 41±5  (1.0) | 40±8 | 2-aminofluorene (10.0) | 994±66  (24.9) |
| TA100 | –S9 | 230±31  (1.0) | 251±10  (1.1) | 229±16  (1.0) | 236±27  (1.0) | 235±12  (1.0) | 238±7  (1.0) | 233±12 | sodium azide (2.0) | 898±67  (3.9) |
|  | +S9 | 237±28  (1.0) | 219±39  (0.9) | 229±34  (1.0) | 244±21  (1.0) | 245±5  (1.0) | 253±7  (1.1) | 234±27 | 2-aminofluorene (10.0) | 882±57  (3.8) |
| TA102 | –S9 | 345±19  (1.0) | 355±10  (1.0) | 354±16  (1.0) | 351±15  (1.0) | 354±23  (1.0) | 350±20  (1.0) | 352±17 | Fenaminosulf (50.0) | 995±22  (2.8) |
|  | +S9 | 344±5  (1.1) | 348±12  (1.1) | 344±12  (1.1) | 345±20  (1.1) | 342±14  (1.0) | 341±17  (1.0) | 326±22 | 1,8-dihydroxyanthraquinone (50.0) | 1065±119  (3.3) |
| TA1535 | –S9 | 14±1  (1.1) | 14±4  (1.1) | 12±2  (0.9) | 11±4  (0.8) | 13±1  (1.0) | 13±3  (1.1) | 13±4 | sodium azide (2.0) | 453±55  (35.8) |
|  | +S9 | 13±2  (0.9) | 14±2  (1.0) | 15±2  (1.0) | 14±3  (1.0) | 15±3  (1.0) | 13±2  (0.9) | 14±2 | cyclophosphamide monohydrate (100.0) | 547±79  (38.1) |

**Ames test of superoxide dismutase.**
